# Small-Pox—Vaccination and Revaccination—False Ideas

**Published:** 1872-07

**Authors:** 


*Small-Pox—Vaccination and Revaccination—False Ideas.*

The following notes regarding vaccination and revaccination have been put together for the purpose of showing how false many of the prevailing ideas on the subject are, and saving time and trouble to those who are engaged in trying to find out the real truth regarding it.

It appears now to be fully acknowledged by medical men—

1. That vaccination, though it greatly lessens the susceptibility of taking small-pox, does not render the reception of it in after years altogether impossible.

2. That vaccination in most cases greatly modifies the character of the small-pox eruption and lessens the severity of the attack.

3. That revaccination gives an absolute (?) immunity from small-pox.

The reasons for coming to these conclusions are as clear as they possibly can be, and there cannot be the smallest allowance made for people who willfully oppose such arguments as the following:

Dr. A. C. C. De Renzy, Sanitary Commissioner in the Punjab says: "In this Province, with a population of 18,000,000, the deaths from small-pox are never less than 20,000 a year. In 1869 they numbered 53,195." In England the annual average mortality does not exceed 5,000, though previous to the introduction of vaccination it was quite as high as in the Punjab.

The facts, again, concerning vaccination in Scotland and Ireland, supplied to Dr. Anstie by Dr. Seaton, of the Privy Council, speak for themselves as plainly as facts possibly can do.

In the former country it appears there was no vaccination act prior to 1863, and the average yearly deaths from small-pox in twelve years, 1853–64, were 1,054. In 1865, '66, '67, and '68, they were respectively 175, 200, 124, and 25.

In Ireland vaccination was not compulsory before 1863, and in the periods of 1830–40, 1840–50, and 1850–60, the respective annual average mortalities were 5,800, 3,827, and 1,272. In the years 1864, '65, '66, '67, '68, they were respectively 854, 337, 187, 20, and 19. In the first quarter of 1869, again, there were only three deaths, and in the second none. Let any one who is skeptical as regards the advantages to be derived from vaccination pass in review the valuable evidence of Mr. Marson, at the small-pox hospital, London. In 5,000 cases of post-vaccinal small-pox, under observation from 1836 to 1855, it appears that there were

35 per cent. of deaths among those that were unvaccinated; 25.57 among those that stated they had been vaccinated, but exhibited no cicatrix. Among those that had 1 cicatrix, the number of deaths per cent. was 7.73; 2 cicatrices, 4.70; 3 cicatrices, 1.95; 4 or more cicatrices, 0.55.

The percentage of deaths among those that had well-marked cicatrices was 2.52, and 8.25 among those who had badly-marked cicatrices. Among those who had small-pox previously, it was 19.

In Pinchbeck, Lincolnshire, with a population of about 3,000 inhabitants, only one death has occurred from small-pox during the last thirty years, and this was in the case of an unvaccinated person. The medical officer of the district has three times received the government grant for efficient vaccination. Dr. Seaton, in his evidence before the Select Committee, stated that vaccination had the effect of reducing the mortality of children under five years of age.

In Scotland, the infantile mortality has been reduced from 70 and 80 per cent. to 55. In Greenock, the mortality under five years of age has been reduced to 36 per cent., and in Glasgow to 28 per cent.

In the "Lancet" of the 8th of April, 1864, the following statement occurred: "Not a single revaccinated case has been admitted into the small-pox hospital at Homerton, and no death of a vaccinated person has occurred under seventeen." "This," as the editor remarked, "shows the protective power of even imperfect vaccination up to puberty, and the necessity for revaccination at this time."

The strongest case one can advocate in support of revaccination, is the fact that not a single nurse has died at the small-pox hospital for the last thirty years, in spite of the infection to which they are constantly exposed. As regards the lymph fit for the purpose of vaccination, a good deal of doubt seems to exist. Some say that carefully-selected matter from revaccination cases is as sure in its effects as that taken from primary vesicles; others, that secondary vaccine matter is of very little use, and that only that taken from the arms of infants should be used; while others assert that the lymph taken from the arms of children has become deteriorated by passing through so many systems, and that a fresh supply should be obtained from the original source. Now, with regard to the use of secondary matter, there can be no doubt that it is capable of setting up the same constitutional disturbance, and producing the same kind of vesicles, as lymph taken from primary cases, but still it not infrequently happens that the vesicles are unduly hastened or otherwise irregular in their development. And from a review of the facts mentioned in the "Lancet," 29th of July, 1871, by Dr. Barbour, of the Stockwell Fever Hospital,

I would say that the amount of protection to be gained from its use is very small, and that it should be employed under very exceptional circumstances; where none other for the time being can be obtained, and revaccination is imperatively demanded, Bryce's test should certainly be employed, in order to see whether it has efficiently performed the purpose for which it was intended or not; in other words, lymph should be again inserted into the arm a few days after the first vaccination. If both vesicles maturate and also die away at the same time, then the first vaccination may be considered to have produced the desired effect; but if the second vesicle goes through all the stages of the primary vesicle, then the first operation has been a mere local affection, and has really exercised no protective influence whatever. The following case occurred in my own experience:

A lady who had apparently been successfully vaccinated with secondary lymph, was one month subsequently revaccinated with primary. The resulting vesicles were as perfect in every respect, and went through exactly the same course as those occurring from a regular primary vaccination.

This was a proof, then, that the first operation produced a local effect only, and that it was incapable of affording protection against the infection of small-pox. In answer to those who affirm that vaccine matter loses its effect by constant use, I would say, for the same reason, the poison of fever ought to become less virulent and infectious each succeeding year; but this is not the case, for though, owing to altered atmospheric influence, prevailing epidemics may, for a time, die out, they soon return when the conditions are again favorable, with all their former activity. But, in addition to this, let us see what opinion Jenner held upon the subject.

After a careful watching of vaccination for upwards of twenty years or more, during which time lymph had been successfully transferred from subject to subject, he came to the conclusion that it underwent no change whatever in its qualities. Marson, Creely, and others also, whose experience is very great, have proved, so far as such matters admit of proof, that vaccine lymph does not lose any of its prophylactic power by a continued transit through successive subjects. When lymph degenerates in transmission, it is invariably due either to want of proper care in the selection of subjects or to inattention to certain details essential to successful vaccination. But let us now, for a moment, consider the evidence of those who have experimented with lymph taken directly from the heifer.

During the siege of Paris, Dr. Quinquand had all successful cases with the human lymph, but only one-third with heifers'.

Dr. Thevenot, with calf vaccine, had only two successful cases out of twenty-one.

Of thirty-two surgeons in Paris who sent in their reports, one says that vaccine from the calf became better after passing through the systems of three or four different children, though bad and difficult to introduce for the first time. The rest (thirty-one) agree that vaccination from the calf was provokingly unsuccessful, succeeding at the very utmost only in a fourth of the children vaccinated directly. Of sixteen others who tried the calf virus, thirteen failed completely.

Dr. Gaillard, who succeeded 170 times out of 283 with calf vaccine, was successful in 2,740 times out of 2,856 with Jennerian vaccine.

The next question, about which some amount of uncertainty prevails, is as to whether syphilis and other diseases can be conveyed by vaccination. Mr. Hutchinson has lately brought forward a series of cases to prove that syphilis is capable of being so conveyed, and, to say the least, they certainly appear to wear a very suspicious aspect. Still, it must be remembered that in 1857, Mr. Simon (medical officer of the Board of Health), addressed a series of questions upon this very subject to a large number of medical men, both in this and other countries, and received answers from no less than 539, with scarcely an exception, entirely in the negative. They declared that syphilis could not be conveyed by means of true vaccination; but they pointed out that, by gross carelessness, it might be inoculated instead of vaccine. The direct experiments, moreover, of Cullerier and others with mixtures of syphilitic matter and vaccine, and vaccine matter taken from persons suffering from constitutional syphilis, are most powerful arguments against the idea that syphilis is able to be transmitted by means of lymph taken out of a true Jennerian vesicle. To show that two poisons cannot be present in a true Jennerian vesicle, lymph may be taken from a vesicle developed in a person who has been vaccinated too late to prevent small-pox, and used without the slightest hesitation for vaccinating another child.

It would certainly appear, from these facts, that vaccination as such can convey syphilis with it through a syphilized lancet, or blood taken up with lymph from a syphilitic infant, may cause it to break out in persons subsequently operated upon. It might, nevertheless, be well to do as Mr. Hutchinson suggests—viz., to avoid taking lymph from first-born children, and take it only from second or later children, in families of which the oldest has enjoyed good health.

As regards the idea of scrofula being conveyed by vaccination, there can be no doubt, as the "Lancet" says, "that it is a mistake. Its development is, on the contrary, greatly prevented, inasmuch as small-pox, by weakening the system, was often the occasion of scrofulous and tubercular disease. Again, when skin eruptions are occasioned by vaccination, the fault is not necessarily with the matter, but with the constitution of the child vaccinated, which cannot bear even the slight disturbance of vaccination with impunity; and *a fortiori* cannot bear the destructive disturbance of small-pox, which is the almost certain alternative."

Among the instructions lately issued by the Lords of the Privy Council are to be noticed the following:

"Never take lymph from cases of revaccination. Never use or furnish lymph which has the slightest admixture of blood. Take lymph only from well-characterized, uninjured vesicles, at the stage (one day week after the vaccination) when they are fully formed and plump, but with no perceptible commencement of areola. Take lymph only which, as it issues from the vesicle, is perfectly clear and transparent, and none which is at all thin and watery. Never squeeze or drain any vesicle. From such a vesicle as vaccination by puncture commonly produces do not, under ordinary circumstances, take more lymph than will suffice for the immediate vaccination of five subjects or for charging several ivory points, or for filling three capillary tubes; and from larger or smaller vesicles take only in proportion to their size. Be careful never to transfer blood from the subject you vaccinate to the subject from whom you take the lymph. Note any case wherein the vaccine vesicle is unduly hastened or otherwise irregular in its development; and if similar results occur in other cases vaccinated with the same lymph, desist at once from employing it. Change the lymph if on the day week after vaccination the vesicles are not entirely free from areola. Keep the lancet and other instruments scrupulously clean, and do not use them for other surgical operations. Cleanse the instrument used thoroughly after one operation before proceeding to another.

"Except so far as any immediate danger of small-pox may require, vaccinate only such subjects as are in good health. As regards infants, ascertain that there is not any febrile state, nor any irritation of the bowels, nor any unhealthy state of skin—especially no chafing or eczema behind the ears, or the groin, or elsewhere in folds of skin. Do not, except of necessity, vaccinate in cases where there has been recent exposure to the infection of measles or scarlatina, nor where erysipelas is prevailing in or about the place of residence.

"Take lymph only from subjects who are in good health, and, as far as you can ascertain, of healthy parentage, preferring children whose families are known to you, and who have elder brothers and sisters of undoubted healthiness. Carefully examine as to skin disease and signs of hereditary syphilis."

There can be no doubt that the present epidemic has had the effect of causing several disputed points to be finally set at rest, and among these, the question as to whether it was right to vaccinate women who were pregnant. The old idea was that vaccine, being a poison similar to that of small-pox, would cause abortion to take place; but this has been contradicted by all the first obstetricians. Next it was thought unsafe to vaccinate children much under six weeks, whereas the medical officer of the Privy Council has distinctly advised that children who are exposed to the influence of the small-pox poison should be vaccinated within a week of their birth.

It was also supposed to be dangerous to revaccinate elderly people, but this has been shown to be incorrect. There can be no question that grown-up people suffer, generally speaking, more from vaccination than children, just as they do when attacked by measles, whooping cough, etc., and this, I believe, has given rise to the idea of vaccination affecting people worse during what is called a varioloid state of atmosphere.

The materials used for collecting lymph are—first, ivory points; second, glasses; third, capillary tubes. The last named are the best, inasmuch as the lymph is always kept in a fresh state.

The methods employed for vaccination are—first, puncturing; second, scratching; third, blistering.

The last mentioned (introduced by Mr. Ellis, of London,) is supposed to render the absorption of the lymph more certain, but it most undoubtedly entails a greater amount of trouble, and I cannot say, from my own experience, that it insures a greater amount of success than either puncturing or scratching when carefully done. In a few instances I have seen very bad arms result from this method. The effects produced by revaccination are not, generally speaking, the same as those which exhibit themselves after primary vaccination. As far as I can see myself the effect of the vaccine manifests itself in three different ways:

1. There may be a perfect vesicle passing through all the different stages, showing that the protective effects of the first vaccination have entirely passed away.

2. There may be a scab formed, but no distinct vesicle, showing that the protective effects of the first vaccination only partially remain.

3. There may be only slight redness produced, showing that the protective effects of the first vaccination remain perfect, or nearly so.

In all cases, I think, where there is not a perfect vesicle, revaccination should be tried again, in order that the operation may not hereafter from carelessness fall into disrepute.

In order that the general public may learn the benefits resulting from vaccination and revaccination, all possible information should be afforded them on the subject, and medical men should be particularly anxious not to let any discredit fall upon the operation from the want of proper care. Once let the public be fully convinced of the fact that they cannot possibly receive harm from the inoculation of vaccine matter, and we may have the satisfaction of seeing small-pox in time banished from our shores.—*Edinburgh Medical Journal.*

